# Simultaneous total occlusion due to spasm of 2 main coronary arteries: A case report

**DOI:** 10.1097/MD.0000000000032656

**Published:** 2023-01-13

**Authors:** Yajing Wang, Ganggang Si, Xiangbing Li, Jianjun Li, Ruxia Zhang, Sancong Pan

**Affiliations:** a Department of Cardiology, Jincheng People’s Hospital, Jincheng, China; b Department of Cardiology, Jincheng Hospital Affiliated to Changzhi Medical College, Jincheng, China.

**Keywords:** acute myocardial infarction (AMI), coronary artery spasm (CAS), diltiazem, emergency coronary angiography (CAG), total occlusion

## Abstract

Coronary artery spasm (CAS) defined by severe reversible focal or diffuse vasoconstriction, plays an essential role in the pathophysiology of acute coronary syndrome but is not very common in the clinic. Due to its transience, it is difficult to diagnose CAS directly, many patients are diagnosed by clinical experience. Here, we report the diagnosis and treatment of a rare case of simultaneous total occlusion due to spasm of the two main coronary arteries by coronary angiography (CAG).

**Patient concerns:**

A 47-year-old man with no medical history was admitted to our emergency room complaining of sudden-onset chest pain lasting 3 hours. Emergency CAG showed total occlusion of the proximal left anterior descending artery and right coronary artery.

**Diagnoses:**

Acute myocardial infarction caused by CAS was diagnosed, according to CAG findings and test results.

**Interventions:**

Intracoronary injection of nitroglycerin and anti-spasm medication.

**Outcomes:**

The patient was discharged on the 8th day with complete resolution of symptoms and normalization of the electrocardiography findings. No ischemic events occurred during a follow-up for 27 months.

**Lessons:**

This case highlights the significance of identifying CAS in patients with acute myocardial infarction and avoiding blind interventional stent therapy, which requires closer attention from clinicians.

## 1. Introduction

Coronary artery spasm (CAS), which is a sudden and reversible vasoconstriction that decreases the lumen of normal or atherosclerotic coronary arteries influencing myocardial blood flow, is characterized by ischemia with no obstructive coronary artery.^[1]^ Despite its transient nature, patients with CAS may present with angina or life-threatening heart events, such as acute myocardial infarction (AMI), lethal arrhythmias, and even sudden death, especially in the case of multivessel involvement.^[2]^ To the best of our knowledge, some cases of CAS have been reported, however simultaneous total occlusion caused by spasm of the 2 main coronary arteries has not been previously reported.

## 2. Case presentation

A 47-year-old man with no significant medical history and no prior complaints of chest pain was admitted to the emergency department with sudden-onset chest pain characterized by squeezing in the left parasternal area that persisted for 3 hours without precipitating factors. He had a smoking history of 20 years, and no family history of coronary artery disease.

**Figure 1.**
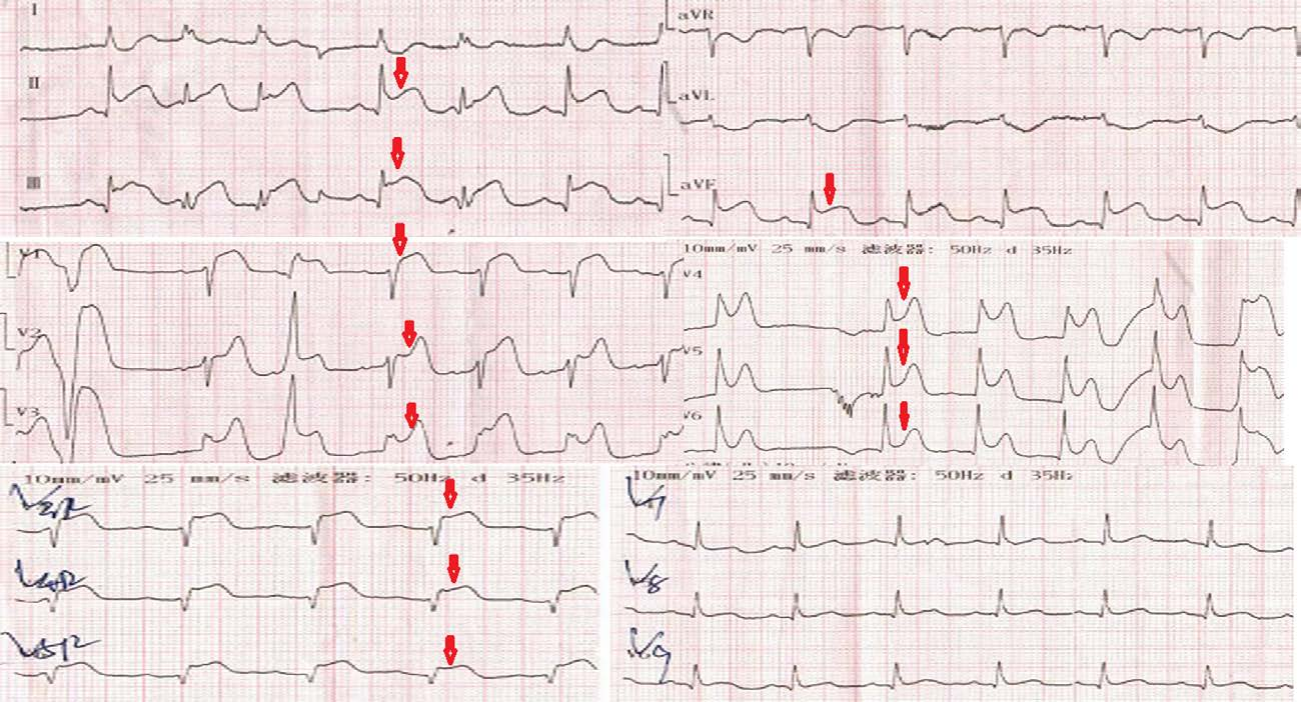
ECG findings on admission revealing widespread ST-segment elevation (red arrow: ST-segment elevation at the anterior, inferior, and right precordial leads). ECG = electrocardiography.

On admission, his heart rate was 73 beats/min, blood pressure was 101/78 mm Hg, and physical examination was negative. Electrocardiography (ECG) revealed sinus rhythm with ST-segment elevation in the widespread anterior, inferior, and right precordial leads (V1-V6, II, III, avF, and V3R–V5R) and reciprocal ST-segment depression in the lateral leads (I and aVL) (Fig.1). Hypersensitive cardiac troponin I test was negative.

**Figure 2.**
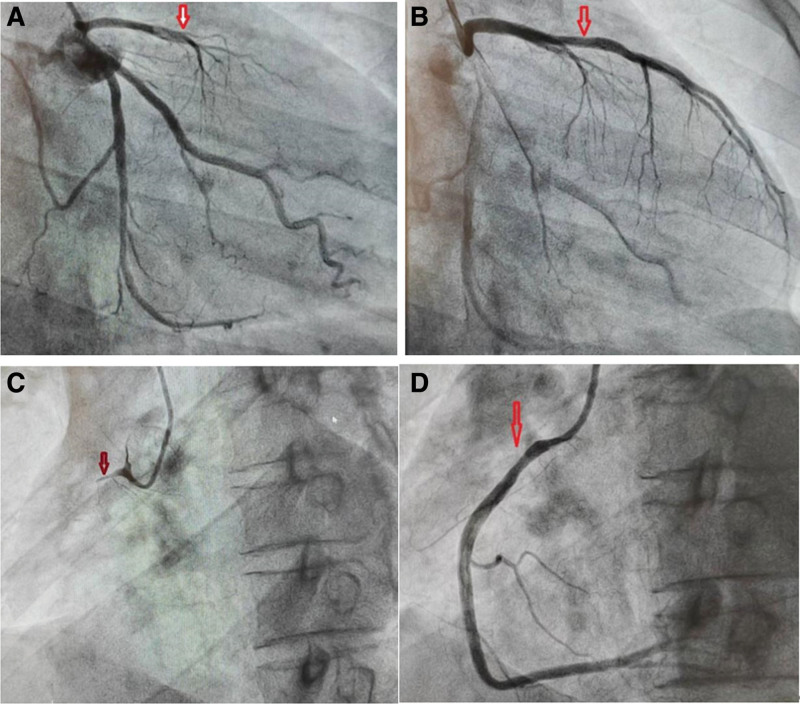
The emergency coronary angiogram demonstrates simultaneous occlusion by 2 main coronary arteries. Panel (A) the proximal segment of the anterior descending artery (LAD) was completely occluded. Panel (B) the original occlusion disappeared after intracoronary injection of nitroglycerin. Panel (C) the proximal segment of the right coronary artery (RCA) was completely occluded. Panel (D) the original occlusion disappeared after intracoronary injection of nitroglycerin. There was no significant stenosis in the arteries.

Emergency coronary angiography (CAG) was performed (Fig.2), and demonstrated simultaneous complete occlusion of the proximal left anterior descending artery (LAD) and proximal right coronary artery (RCA). We decided to perform percutaneous coronary intervention for him. Surprisingly, LAD became completely normal after intracoronary administration of nitroglycerin 200 µg in it, and the same phenomenon occurred in the RCA. Organic coronary stenosis was not observed. The patient’s symptoms were generally alleviated. Electrocardiographic monitoring revealed recovery of ST-segment elevation. During angiography, low blood pressure and slow heart rate occurred, which returned to normal after intravenous injection of vasoactive drugs (epinephrine, atropine, dopamine, and norepinephrine).

**Figure 3.**
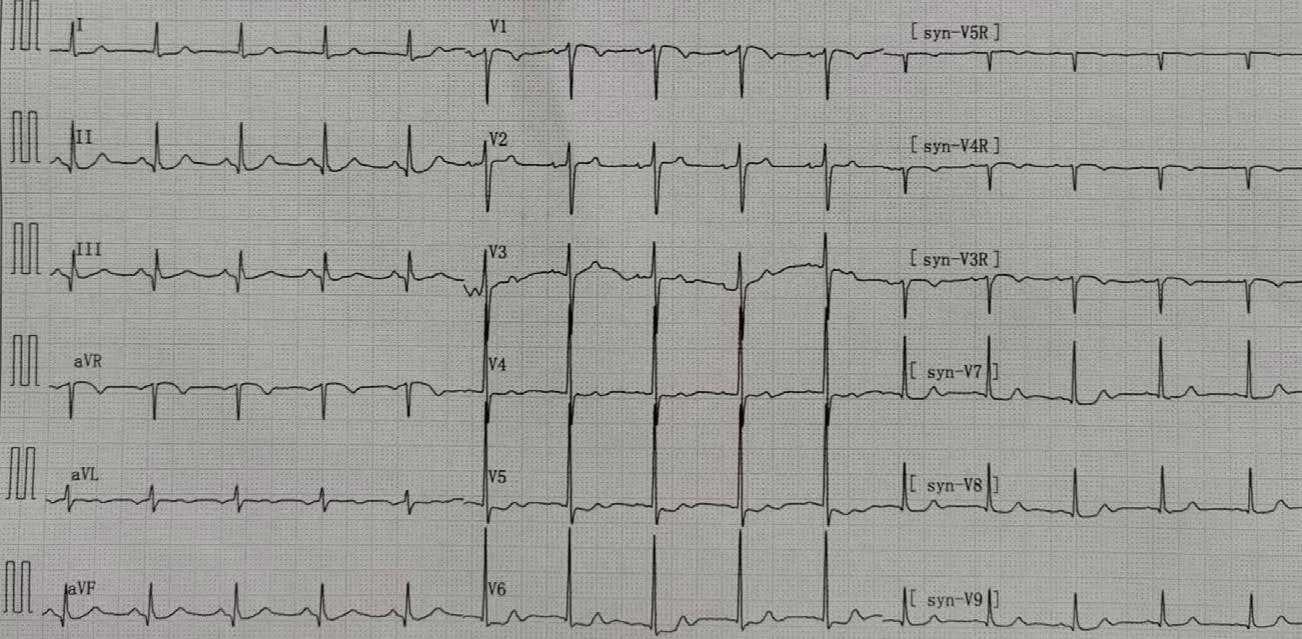
The ECG of the patient on the next day, he had no chest discomfort. ECG = electrocardiography.

Then, the patient was monitored in the Cardiac Care Unit. The ECG in the Cardiac Care Unit showed an ST-segment almost back to the baseline (Fig.3). Laboratory result showed hypersensitive cardiac troponin I levels of 50 ng/mL at the peak (normal range 0–0.06 ng/mL), low-density-lipoprotein cholesterol (LDL-C) level was 3.36 mmol/L (normal range 1.0–3.1 mmol/L). While other laboratory tests results were within normal ranges. Echocardiography presented a new inferior left ventricular wall and post-ventricular septum hypokinesis, with an estimated left ventricular ejection fraction of 60%.

Based on the medical history, CAG findings, and test results, the diagnosis of AMI was ultimately made with simultaneous total occlusion due to persistent spasm of the 2 main coronary arteries. Lifestyle changes were recommended, including cessation of smoking and long-term medicines were administrated including diltiazem, isosorbide mononitrate, aspirin, clopidogrel, and atorvastatin. During hospitalization, the patient did not have any discomfort and no arrhythmias occurred. On the 8th day of admission, the patient was discharged with complete resolution of symptoms and normalization of the ECG findings. During regular outpatient follow-up for 27 months, no major adverse cardiac events occurred, and no angina was reported by him.

## 3. Discussion

CAS is uncommon in patients with ischemic heart disease irrespective of racial, genetic, and geographic variations.^[1]^ The concept of CAS was first proposed by Prinzmetal et al by describing non-exertional angina occurring during regular daily activities or at rest,^[3]^ which was later confirmed and demonstrated in several experimental studies. There have been reported many cases of CAS due to different situations, for example, Piel et al^[4]^ and Bai L et al^[5]^ reported extreme ST-segment elevations due to CAS, Xin Xie et al^[6]^ reported severe CAS during left atrial appendage closure plus catheter ablation for atrial fibrillation, but simultaneous total occlusion of the 2 main coronary arteries caused by CAS has not been reported, therefore, we reported this case in which a patient suffered from persistent LAD and RCA spasm, even occlusion, who eventually presented with myocardial infarction.

The manifestations of CAS according to the coronary segments and frequencies of angina onsets vary vastly from asymptomatic to angina, AMI, ventricular arrhythmias, and sudden cardiac death.^[7]^ Unlike vasovagal symptoms, CAS occurs regularly at rest and early in the morning, between midnight and 5 am.^[8]^

The main risk factors for CAS are smoking, age, LDL-C level, hypertension, diabetes mellitus, and high-sensitivity C-reactive protein level.^[9]^ CAS is more common in males than females aged between 40 and 70 years.^[1,10]^ Smoking was considered to be involved in the onset of CAS, which would trigger vascular injury, a decline in the partial pressure of oxygen in the myocardium and coronary arterial flow.^[11]^ There was a positive correlation between coronary arterial contraction and LDL-C and total cholesterol levels during CAS.^[12]^ High concentration of LDL-C can affect the prognosis of patients with CAS. The coexistence of several risk factors can strengthen the ability to induce CAS, which may lead to a vicious circle. In the present case, the patient was a 47-year male with increased LDL-C levels and heavy smoking, which may explain the onset of CAS.

The underlying pathophysiology of CAS is not clearly understood, which could have multifactorial implications on the autonomic nervous system, inflammation, endothelial dysfunction, atherosclerosis, thrombosis and hyperreactivity, etc.^[1]^ The potential mechanism of CAS in this case may involve the following aspects: the first is the patient’s risk factors of smoking and high LDL-C levels, which would contribute to chronic inflammation and atherosclerosis, resulting in hyperreactivity and increasing the risk of CAS. In addition, the autonomic nervous system plays an important role; the parasympathetic nervous system is supported by the frequent occurrence of CAS at midnight or rest correlating with the highest vagal activity.^[13]^ Our patient had angina at rest, confirming this hypothesis.

The most reliable method for diagnosing coronary vasospasm is the provocation test adjunctive to CAG,^[14]^ which is rarely performed owing to serious side effects, although it has been confirmed to be safe and has relevant prognostic implications.

Coronary imaging can be used to detect CAS specific findings. Intravascular ultrasound can identify small lesion plaque volume and burden, diffuse coronary intimal thickening, and different plaque components. Nevertheless, optical coherence tomography can delineate detailed structural changes in the coronary arteries of patients with CAS. In our case, we confirmed CAS in the LAD and RCA by CAG. However, we were unable to perform intravascular ultrasound or optical coherence tomography to identify the possible mechanism due to the cost, state of the patient, and invasive method.

The treatment of CAS mainly involves lifestyle changes (such as smoking cessation), antispasmodic medications and other cardiovascular protective therapy.^[2]^ Nitrates remain a critical treatment to relieve acute attacks of CAS,^[15]^ which can relax the smooth muscles and subsequently relieve spasmodic pain. However, owing to drug-related issues, long-term use of nitrates is not recommended unless it is effective. Long-acting calcium channel blockers (CCBs) are beneficial to myocardial oxygen supply in patients with CAS. A near-complete reduction in CAS recurrence is observed with nondihydropyridine CCBs (e.g., diltiazem) rather than with dihydropyridine CCBs.^[16]^ Studies have documented a positive role for statins in suppressing CAS episodes and decreasing the risk of recurrence by improving endothelia function.^[17,18]^ Antiplatelet therapy plays a controversial role. Stenting is only recommended in patients with CAS and significant stenosis of the coronary artery.^[19]^ Implantable cardiac defibrillator therapy is limited to life-threatening situations defined by cardiac arrest survivors and episodes of ventricular arrhythmia.^[19,20]^ CAS can lead to lethal arrhythmia and silent angina. However, implantable cardiac defibrillator indications remain unclear and are recommended for secondary prevention. For this patient, with the above medical treatment, no major adverse cardiovascular events occurred.

## 4. Conclusion

CAS is a complex multifactorial disease that can lead to serious complications, which often present as local or whole arteries, and occurs in 2 or 3 arteries at different times or simultaneously in the same patient. However, simultaneous total occlusion caused by spasms of the 2 main coronary arteries, which is a severe or lethal condition, is rare. Physicians should consider this condition to better recognize it early and start appropriate treatment, which would prevent the aggravation of spasm attacks and improve long-term outcomes.

## Author contributions

**Conceptualization:** Yajing Wang, Sancong Pan, Ganggang Si.

**Data curation:** Xiangbing Li, Ruxia Zhang.

**Formal analysis:** Yajing Wang.

**Funding acquisition:** Sancong Pan.

**Project administration:** Yajing Wang, Sancong Pan, Gangang Si.

**Resources:** Jianjun Li, Xiangbing Li, Ruxia Zhang.

**Software:** Yajing Wang.

**Supervision:** Sancong Pan.

**Validation:** Sancong Pan.

**Visualization:** Ruxia Zhang, Yajing Wang.

**Writing – original draft:** Yajing Wang, Sancong Pan, Ganggang Si.

**Writing – review & editing:** Yajing Wang, Sancong Pan.

## References

[1] Matta A Bouisset F Lhermusier T. Coronary artery spasm: new insights. J Interv Cardiol. 2020;2020:15894586–10. 10.1155/2020/5894586 PMC7245659 32508542

[2] Slavich M Patel RS. Coronary artery spasm: current knowledge and residual uncertainties. Int J Cardiol Heart Vasc. 2016;23:47–53. 10.1016/j.ijcha.2016.01.003 PMC5462634 28616515

[3] Kennamer M Merliss R Wada R. A variant form of angina pectoris. Am J Med. 1959;27:375–88. 14434946 10.1016/0002-9343(59)90003-8

[4] Piels M Faes T Vainer J. Extreme ST-segment elevations in seemingly no significant angiographic coronary artery abnormalities: a case report. BMC Cardiovasc Disord. 2019;19:28. 30696424 10.1186/s12872-019-1010-x PMC6350352

[5] Bai L Chen F Peng Y. Widespread ST-segment elevation due to diffuse coronary artery spasm: a case report. Ann Noninvasive Electrocardiol. 2021;26:e12877. 34250702 10.1111/anec.12877 PMC8411782

[6] Xin X Zijun C Yu L. Severe coronary artery spasm during left atrial appendage closure plus catheter ablation for atrial fibrillation: case presentation. BMC Cardiovasc Disord. 2022;22:38. 35148671 10.1186/s12872-022-02483-2 PMC8832689

[7] Nakamura M Takeshita A Nose Y. Clinical characteristics associated with myocardial infarction, arrhythmias, and sudden death in patients with vasospastic angina. Circulation. 1987;75:1110–6. 3568322 10.1161/01.cir.75.6.1110

[8] Kusama Y Kodani E Nakagomi A. Variant angina and coronary artery spasm: the clinical spectrum, pathophysiology, and management. J Nippon Med Sch. 2011;78:4–12. 21389642 10.1272/jnms.78.4

[9] Libby P Bornfeldt KE Tall AR. Atherosclerosis. Circ Res. 2016;118:531–4. 26892955 10.1161/CIRCRESAHA.116.308334 PMC4762065

[10] Hung MY Hsu KH Hung MJ. Interactions among gender, age, hypertension and C-reactive protein in coronary vasospasm. Eur J Clin Invest. 2010;40:1094–103. 20718850 10.1111/j.1365-2362.2010.02360.x

[11] Brunner H Cockcroft JR Deanfield J. Endothelial function and dysfunction. Part II: Association with cardiovascular risk factors and diseases. A statement by the working group on endothelins and endothelial factors of the European society of hypertension. J Hypertens. 2005;23:233–46. 15662207 10.1097/00004872-200502000-00001

[12] Nedeljkovic MA Ostojic MC Beleslin BD. Ergonovine-induced changes of coronary artery diameter in patients with nonsignificant coronary artery stenosis:relation with lipid profile. Herz. 2007;32:329–35. 17607540 10.1007/s00059-007-2831-4

[13] Waters DD Miller DD Bouchard A. Circadian variation in variant angina. Am J Cardiol. 1984;54:61–4. 6741840 10.1016/0002-9149(84)90304-7

[14] Kurita T. Coronary artery spasms and ST-segment elevation during catheter ablation of pulmonary vein isolation- cause, mechanism, and management. Circ J. 2021;85:272–4. 33504713 10.1253/circj.CJ-20-1238

[15] Guidelines for diagnosis and treatment of patients with vasospastic angina (Coronary Spastic Angina) (JCS 2013). Circ J. 2014;78:2779–801. 25273915 10.1253/circj.cj-66-0098

[16] Rodr´iguez-Mañero M Oloriz T Le polain de Waroux J. Long-term prognosis of patients with life-threatening ventricular arrhythmias induced by coronary artery spasm. EP Europace. 2018;20:851–8. 28387796 10.1093/europace/eux052

[17] Piao ZH Jeong MH Li Y. Benefit of statin therapy in patients with coronary spasm-induced acute myocardial infarction. J Cardiol. 2016;68:7–12. 26584973 10.1016/j.jjcc.2015.09.013

[18] Ishii M Kaikita K Sato K. Impact of statin therapy on clinical outcome in patients with coronary spasm. J Am Heart Assoc. 2016;5:003426. 10.1161/JAHA.116.003426 PMC4889205 27207970

[19] Beltrame JF Crea F Kaski JC. International standardization of diagnostic criteria for vasospastic angina. Eur Heart J. 2017;38:2565–8. 26245334 10.1093/eurheartj/ehv351

[20] Sueda S Kohno H. Optimal medications and appropriate implantable cardioverter-defibrillator shocks in aborted sudden cardiac death due to coronary spasm. Intern Med. 2018;57:1361–9. 29321418 10.2169/internalmedicine.8796-17 PMC5995710

